# Comparison of Transperitoneal and Retroperitoneal Robotic Partial Nephrectomy for Patients With Complete Upper Pole Renal Tumors

**DOI:** 10.3389/fonc.2021.773345

**Published:** 2022-01-25

**Authors:** Liangyou Gu, Wenlei Zhao, Junnan Xu, Baojun Wang, Qiang Cheng, Donglai Shen, Yundong Xuan, Xupeng Zhao, Hongzhao Li, Xin Ma, Xu Zhang

**Affiliations:** Department of Urology, the Third Medical Centre, Chinese PLA General Hospital, Beijing, China

**Keywords:** kidney neoplasms, upper pole, partial nephrectomy, robotics, outcome

## Abstract

**Objectives:**

We compared the outcomes of transperitoneal robotic partial nephrectomy (TRPN) and retroperitoneal robotic partial nephrectomy (RRPN) for complete upper pole renal masses (1 point for the “L” component of the RENAL scoring system).

**Material and Methods:**

We retrospectively reviewed patients who underwent either TRPN or RRPN from 2013 to 2016. Baseline demographics and perioperative, functional, and oncological results were compared. Multivariable analysis was performed to identify factors related to pentafecta achievement (ischemia time ≤25 min, negative margin, perioperative complication free, glomerular filtration rate (eGFR) preservation >90%, and no chronic kidney disease upstaging).

**Results:**

No significant differences between TRPN *vs.* RRPN were noted for operating time (110 *vs.* 114 min, *p* = 0.870), renal artery clamping time (19 *vs.* 18 min, *p* = 0.248), rate of positive margins (0.0% vs. 3.3%, *p* = 0.502), postoperative complication rates (25.0% *vs.* 13.3%, *p* = 0.140). TRPN was associated with a more estimated blood loss (50 *vs.* 40 ml, *p* = 0.004). There were no significant differences in pathologic variables, rate of eGFR decline for postoperative 12-month (9.0% *vs.* 7.1%, *p* = 0.449) functional follow-up. Multivariate analysis identified that only RENAL score (odd ratio: 0.641; 95% confidence interval: 0.455–0.904; *p* = 0.011) was independently associated with the pentafecta achievement.

**Conclusions:**

For completely upper pole renal masses, both TRPN and RRPN have good and comparable results. Both surgical approaches remain viable options in the treatment of these cases.

## Introduction

Partial nephrectomy (PN) remains the standard treatment for cT1a renal tumors (1, 2) and increasingly being used to manage more complex masses (3). Due to the similar oncologic outcome, faster recovery, and reduced blood loss and wound complications, PN has transitioned from open to the minimally invasive approach. Before the advent of robot surgical system, laparoscopic partial nephrectomy (LPN) has been widely performed. However, continued concern about prolonging renal artery clamping time and the complexity of suturing and excision are obstacles to the use of LPN. In China, robotic partial nephrectomy (RPN) has been more and more widely used in treating renal masses, especially for more complex lesions. Due to the advantaged of robot surgical system, RPN provides improvements in estimated blood loss, ischemia time, and postoperative hospital stay to LPN (4).

RPN can be conducted through transperitoneal or retroperitoneal approach (5). In China, RPN was initially performed exclusively *via* the abdominal approach. Because of the extensive experiences in retroperitoneal LPN (6, 7), we have tried to perform retroperitoneal robotic partial nephrectomy (RRPN) for some patients with posterior renal tumors. Many studies have compared the outcomes between transperitoneal robotic partial nephrectomy (TRPN) and RRPN for patients with renal tumors, including renal masses in different locations (8–10) or only lateral (11) or posterior (12–14) tumors. Recently, a meta-analysis summarizing the results from these studies has been published (15). They found that RRPN can obtain more favorable outcomes than TRPN, including shorter operative time, less estimated blood loss, less minor complications, and shorter hospital stay.

For complete upper pole renal tumors, RPN can be performed by transperitoneal or retroperitoneal approach. The transabdominal approach is familiar to urologists, and adequate operating space to avoid instruments collision. However, because of the proximity of the abdominal organs, it was more difficult to expose renal hilum and tumors. In contrast, the advantage of the retroperitoneal approach is that it reduces morbidity and speeds recovery by avoiding abdominal and unobstructed access to the hilum (14, 16). Hence, we initially compared the perioperative, functional and oncological outcomes of TRPN and RRPN for complete upper pole renal tumors.

## Methods

### Patients

**Figure 1:**
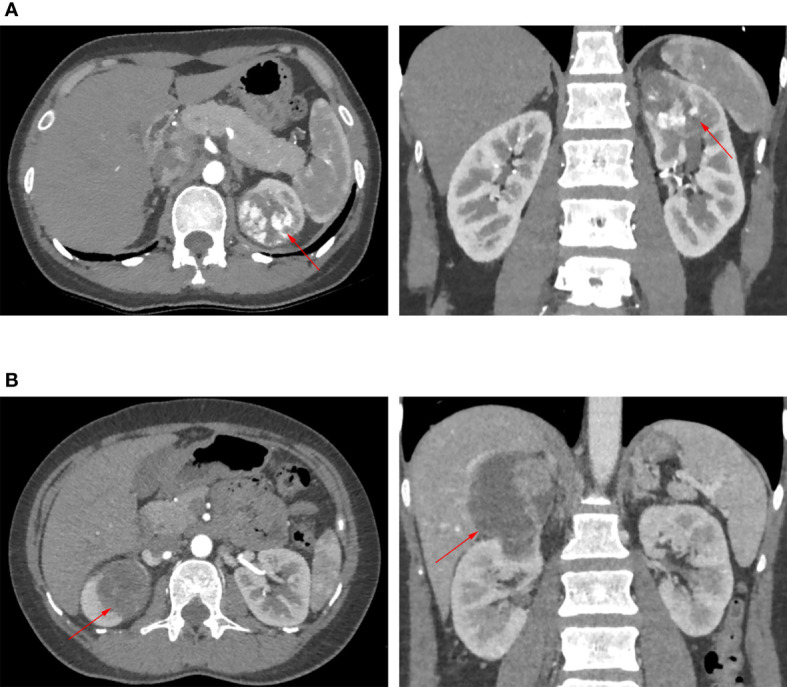
Representative images of CT or MRI. Red arrow points to renal tumor. **(A)** The patient underwent transperitoneal robotic partial nephrectomy. **(B)** The patient underwent retroperitoneal robotic partial nephrectomy.

A prospectively established renal tumor database has been maintained in our hospital. After being approved by the institutional review board, we retrospectively reviewed patients who underwent either transperitoneal robotic partial nephrectomy (TRPN) or retroperitoneal robotic partial nephrectomy (RRPN) for localized renal tumor between 2013 and 2016. The RENAL scoring system was used to assess the complexity of renal tumors based on preoperative CT or MRI (17). Only patients having complete upper pole renal tumors were included in our study. Complete upper pole renal tumor was defined as tumor located in the upper pole and attributed 1 point for the “L” component of the RENAL scoring system (17). Representative images are shown in **Figure 1**. The approach (transperitoneal or retroperitoneal) of robotic partial nephrectomy was decided according to the expertise and preference of the performing surgeons. All surgical procedures were performed by experienced surgeons; they have crossed the learning curve. The renal artery clamping was applied in all cases, and hypothermic ischemic technique was used in some selected cases. After excluding the patients lacking key data, 108 patients were included in the present study, 48 of them underwent TRPN and 60 underwent RRPN.

### Patient Positioning and Trocar Placement

For TRPN, the patient was placed in the 60°–70° lateral decubial position with a gel pad supporting the lateral extension. A pillow was placed under the waist to elevate the waist for maximum flanking extension (**Supplementary Figure S1A**). After pneumoperitoneum was established, a 10-mm incision was made on semilunalis 2-finger breadths above the umbilical and a 12-mm trocar was inserted as the camera trocar. The 8-mm robotic trocar was first inserted into the palm-width position above the camera trocar, and then another 8-mm robotic trocar was inserted 2-finger breadths above and lateral to ASIS, with a palm width distance from the camera trocar. Third robotic trocar can be inserted just above the pubic tubercle on the semilunalis, with a palm width distance from second robotic trocar (**Supplementary Figure S1B**).

For RRPN, patient was positioned to lateral decubitus position with extended flank. A gel cushion was inserted below the waist to elevate the waist and maximize the flank extension (**Supplementary Figure S2A**). A 3-cm transverse incision was made 2 cm above the iliac crest in the midline of the axilla. After the retroperitoneal space was expanded, an 8-mm robotic trocar was inserted from the costal margin of the posterior axillary line to the midpoint of the iliac crest. This trocar is used to insert the second arm. A 12-mm camera trocar was inserted into the 3-cm incision, and the incision was sutured. Under direct vision, the trocar of the first arm was inserted 1–2 cm inside the axillary front, at the same level as the second arm. Insert a 12-mm auxiliary trocar at the midpoint between the first arm trocar and the camera trocar (**Supplementary Figure S2B**).

### Date Collection

The relevant data were extracted from our database. Demographics, disease characteristics, perioperative outcomes, and pathological and renal functional outcomes were compared between TRPN and RRPN. Demographics included age, gender, body mass index (BMI), ASA score, Charlson comorbidity index (CCI) score, presence of diabetes or hypertension, and history of abdominal surgery. Disease characteristics included symptoms, tumor side and size, RENAL score, preoperative serum creatinine, and estimated glomerular filtration rate (eGFR). eGFR was calculated with the CKD-EPI equation (18). Perioperative outcomes embraced operative time, ischemia time, estimated blood loss, blood transfusion, length of hospital stay, and complications. Complications were recorded according to the modified Clavien-Dindo classification system (19, 20). Pathological outcomes included tumor histology, pT stage, Fuhrman grade, and tumor necrosis. All patients were rechecked by a genitourinary pathologist. Renal functional outcomes were evaluated by postoperative 1-day and 12-month eGFR change. Pentafecta also was chosen to be an important outcome, which was defined as negative surgical margin, an ischemia time ≤25 min, no perioperative complications, eGFR preservation >90%, and no CKD upstaging (21). An upgrade to CKD is considered to be an upgrade to stage III, IV, or V and does not include stages I through II. Each patient was followed up regularly after surgery. Details of local recurrence and distant metastasis were recorded.

### Statistical Analysis

For continuous variables, median and interquartile range were used for describing them, and Wilcoxon rank sum test was applied for testing the difference. Fisher’s exact and Pearson’s Chi-square tests were used for categorical variables. Univariable and multivariable logistic regression analyses were used to determine risk factors associated with achievement of pentafecta. Variables were selected for multivariate analysis according to univariate analysis results and clinical experience. Statistical analysis was carried out using R software (version 3.3.1), with significance defined as *p* < 0.05.

## Results

In total, 108 patients were included in the present study, 48 of them underwent TRPN and 60 underwent RRPN. **Table 1** shows demographics and disease characteristics. No significant difference was identified between the TRPN and RRPN groups in terms of demographics (age, gender, BMI, ASA score, CCI score, presence of diabetes or hypertension, history of abdominal surgery), laterality distribution (*p* = 0.702), median tumor size (2.8 *vs.* 3.3 cm, *p* = 0.840), median RENAL score (6 *vs.* 6, *p* = 0.908), median preoperative serum creatinine (69.2 *vs.* 74.2 µmol/L, *p* = 0.969), and median preoperative eGFR (97.3 *vs.* 97.6 ml/min/1.73 m^2^, *p* = 0.951). When classified by the complexity of RENAL score, no significant difference was identified between the TRPN and RRPN groups in terms of low (54.2% *vs.* 55.0%), moderate (45.8% *vs.* 45.0%), and complex (0.0% *vs.* 0.0%, *p* = 1.000). Analyzing the “A” (anterior/posterior) domain of RENAL score, no significant difference was identified between the TRPN and RRPN groups in terms of anterior (25.0% *vs.* 23.3%), posterior (45.8% *vs.* 41.7%), not determined (29.2% *vs.* 35.0%, *p* = 0.853).

**Table 1:** Patients’ demographics and tumor characteristics.

	Overall	TRPN	RRPN	*p*-value
No. patients	108	48	60	
Age [years, median (IQR)]	51 (43–60)	50 (43–60)	53 (45–60)	0.403
Male patients [*n* (%)]	69 (63.9)	31 (64.6)	38 (63.3)	1.000
BMI (kg/m^2^, median [IQR)]	25.5 (23.4–27.5)	24.9 (23.3–26.6)	26.0 (23.4–28.2)	0.451
ASA score [*n* (%)]				
1 and 2	104 (96.3)	45 (93.8)	59 (98.3)	0.321
3 and 4	4 (3.7)	3 (6.3)	1 (1.7)	
CCI score [*n* (%)]				
0–1	97 (89.8)	42 (87.5)	55 (91.7)	0.534
≥2	11 (10.2)	6 (12.5)	5 (8.3)	
Clinical symptoms [*n* (%)]	8 (7.4)	3 (6.3)	5 (8.3)	0.730
Presence of diabetes [*n* (%)]	14 (13.0)	6 (12.5)	8 (13.3)	1.000
Presence of hypertension [*n* (%)]	27 (25.0)	14 (29.2)	13 (21.7)	0.503
Prior abdominal surgery [*n* (%)]	27 (25.0)	11 (22.9)	16 (26.7)	0.823
Solitary kidney [*n* (%)]	2 (1.9)	0 (0.0)	2 (3.3)	0.502
Left tumor [*n* (%)]	52 (48.1)	22 (45.8)	30 (50.0)	0.702
Tumor size [cm, median (IQR)]	3.1 (2.3–4.0)	2.8 (2.2–3.8)	3.3 (2.4–4.3)	0.840
RENAL score [median (IQR)]	6 (5–7)	6 (5–7)	6 (5–7)	0.908
RENAL complexity class				
Low (4–6)	59 (54.6)	26 (54.2)	33 (55.0)	1.000
Moderate (7–9)	49 (45.4)	22 (45.8)	27 (45.0)	
High (10–12)	0 (0.0)	0 (0.0)	0 (0.0)	
Anterior/Posterior aspect [*n* (%)]				
Anterior	26 (24.1)	12 (25.0)	14 (23.3)	0.853
Posterior	47 (43.5)	22 (45.8)	25 (41.7)	
Not determined	35 (32.4)	14 (29.2)	21 (35.0)	
Hypothermic ischemia [*n* (%)]	1 (0.9)	1 (2.1)	0 (0.0)	0.444
Preoperative creatinine [(µmol/L), median (IQR)]	70.7 (61.7–84.8)	69.2 (60.1–84.8)	74.2 (63.5–84.8)	0.969
Preoperative eGFR [(ml/min/1.73 m^2^), median (IQR)]	97.6 (84.4–107.2)	97.3 (85.6–106.0)	97.6 (83.4–107.3)	0.951

TRPN, transperitoneal robotic partial nephrectomy; RRPN, retroperitoneal robotic partial nephrectomy; IQR, interquartile range; BMI, body mass index; ASA, American Society of Anesthesiologists; CCI, Charlson Comorbidity Index; eGFR, estimated glomerular filtration rate.

**Table 2** shows the perioperative results. Patients undergoing TRPN had more estimated blood loss (50 *vs.* 40 ml, *p* = 0.004). There were no significant differences between the TRPN and RRPN in operative time (110 *vs.* 114 min; *p* = 0.870), ischemia time (19 *vs.* 18 min, *p* = 0.248), transfusion rate (2.1% vs. 0.0%, *p* = 0.444), positive margin rate (0.0% *vs.* 3.3%, *p* = 0.502), length of hospital stay (11 *vs.* 11 days, *p* = 0.579), and postoperative complication rate (25.0% *vs.* 13.3%, *p* = 0.140).

**Table 2:** Perioperative outcomes.

Variable	TRPN	RRPN	*p*-value
Operating time [min, median (IQR)]	110 (90–139)	114 (85–140)	0.870
Estimated blood loss [ml, median (IQR)]	50 (50–100)	40 (20–50)	0.004
Renal artery clamping time [min, median (IQR)]	19 (13–24)	18 (11–13)	0.248
Transfusion [*n* (%)]	1 (2.1)	0 (0.0)	0.444
Conversion to radical [*n* (%)]	0 (0.0)	0 (0.0)	1.000
Conversion to open [*n* (%)]	0 (0.0)	0 (0.0)	1.000
Positive surgical margin [*n* (%)]	0 (0.0)	2 (3.3)	0.502
Postoperative hospital stay [day, median (IQR)]	11 (10–13)	11 (10–13)	0.579
Postoperative complications [*n* (%)]	12 (25.0)	8 (13.3)	0.140
Minor	12 (25.0)	7 (11.7)	0.081
Clavien 1	6 (12.5)	3 (5.0)	
Clavien 2	6 (12.5)	4 (6.7)	
Major	0 (0.0)	1 (1.7)	1.000
Clavien 3	0 (0.0)	1 (1.7)	
Clavien 4	0 (0.0)	0 (0.0)	

TRPN, transperitoneal robotic partial nephrectomy; RRPN, retroperitoneal robotic partial nephrectomy; IQR, interquartile range.

**Table 3** shows the pathological, renal, functional, and oncological outcomes. Most patients were proven to be clear cell renal cell carcinoma. There were no significant differences between the TRPN and RRPN in tumor histology (*p* = 0.922), pT stage (*p* = 0.268), Fuhrman grade (*p* = 0.699), tumor necrosis rate (4.2% *vs.* 6.7%, *p* = 0.691). For postoperative renal function, no significant difference was identified between the two groups in 1-day ΔeGFR rate (14.2% *vs.* 17.1%, *p* = 0.581) and 12-month ΔeGFR rate (9.0% *vs.* 7.1%, *p* = 0.449). During the similar follow-up time (63.1 *vs.* 58.5 months, *p* = 0.382), there were no significant differences in local recurrence rate (2.1% *vs.* 1.7%, *p* = 1.000) and distant metastasis rate (2.1% vs. 3.3%, *p* = 1.000).

**Table 3:** Pathological outcomes and follow-up data.

Variable	TRPN	RRPN	*p*-value
Tumor histology [*n* (%)]			
Clear cell RCC	45 (93.8)	54 (90.0)	0.922
Papillary RCC	1 (2.1)	1 (1.7)	
Chromophobe RCC	1 (2.1)	2 (3.3)	
Other types	1 (2.1)	3 (5.0)	
Pathologic stage [*n* (%)]			
T1a	39 (81.3)	43 (71.7)	0.268
T1b	9 (18.8)	17 (28.3)	
Fuhrman grade [*n* (%)]			
Low (1–2)	41 (91.1)	51 (94.4)	0.699
High (3–4)	4 (8.9)	3 (5.6)	
Tumor necrosis [*n* (%)]	2 (4.2)	4 (6.7)	0.691
Postoperative eGFR, median (IQR)			
1-day eGFR (ml/min/1.73 m^2^)	83.5 (69.4–101.1)	84.7 (62.4–101.1)	0.683
1-day % eGFR decline	14.2 (8.7–20.2)	17.1 (6.3–23.7)	0.581
12-month eGFR (ml/min/1.73 m^2^)	89.5 (75.7–103.4)	95.9 (75.9–102.2)	0.683
12-month % eGFR decline	9.0 (4.9–13.2)	7.1 (3.4–13.9)	0.449
Follow-up [months, median (IQR)]	63.1 (58.5–73.9)	58.5 (55.0–62.0)	0.382
Oncological outcomes [*n* (%)]			
Local recurrence	1 (2.1)	1 (1.7)	1.000
Distant metastasis	1 (2.1)	2 (3.3)	1.000

TRPN, transperitoneal robotic partial nephrectomy; RRPN, retroperitoneal robotic partial nephrectomy; RCC, renal cell carcinoma; eGFR, estimated glomerular filtration rate; IQR, interquartile range.

**Table 4** shows pentafecta outcomes between TRPN and RRPN. The two groups had similar pentafecta rates (50.0% *vs.* 55.0%, *p* = 0.699). When pentafecta was classified into individual elements, no significant difference was identified in negative margins (100.0% *vs.* 96.7%, *p* = 0.502), no complication (75.0% *vs.* 86.7%, *p* = 0.140), ischemia time ≤25 min (81.3% *vs.* 86.7%, *p* = 0.596), eGFR recovery to >90% baseline (72.9% *vs.* 75.0%, *p* = 0.828), and CKD upstaging free (100.0% *vs.* 96.7%, *p* = 0.502). **Table 5** shows univariable and multivariable analyses of factors related to pentafecta achievement. In univariable analysis, factors with *p* < 0.10 included tumor size, preoperative eGFR, and RENAL score. These factors together with surgical type were entered into multivariable analysis. The results showed that only decreasing RENAL score [OR, 0.641 (95% CI, 0.455–0.904); *p* = 0.011] was independent risk factor related to pentafecta achievement, but not surgical type (*p* = 0.600).

**Table 4:** Pentafecta analysis comparing TRPN and RRPN.

Outcome	TRPN	RRPN	*p*-value
Negative margins [*n* (%)]	48 (100.0)	58 (96.7)	0.502
No complications [*n* (%)]	36 (75.0)	52 (86.7)	0.140
Ischemia time ≤25 min [*n* (%)]	39 (81.3)	52 (86.7)	0.596
eGFR >90% of preop [*n* (%)]	35 (72.9)	45 (75.0)	0.828
No CKD upstaging [*n* (%)]	48 (100.0)	58 (96.7)	0.502
“Pentafecta” [*n* (%)]	24 (50.0)	33 (55.0)	0.699

TRPN, transperitoneal robotic partial nephrectomy; RRPN, retroperitoneal robotic partial nephrectomy; eGFR, estimated glomerular filtration rate; CKD, chronic kidney disease.

**Table 5:** Univariable and multivariable analyses for factors associated with achieving pentafecta.

Variable	Univariate		Multivariate	
	OR (95% CI)	*p*-value	OR (95% CI)	*p*-value
Age	1.005 (0.956–1.070)	0.639		
BMI	0.975 (0.866–1.099)	0.682		
Sex (male vs. female)	1.290 (0.587–2.834)	0.526		
Diabetes	0.880 (0.286–2.706)	0.823		
Hypertension	0.952 (0.398–2.276)	0.911		
CCI (≥2 vs. 0–1)	0.721 (0.206–2.522)	0.609		
ASA score (3 + 4 vs. 1 + 2)	2.778 (0.280–27.585)	0.383		
Prior abdominal surgery	0.781 (0.326–1.868)	0.578		
Tumor laterality (right vs. left)	0.793 (0.372–1.692)	0.549		
Tumor size	0.672 (0.485–0.933)	0.017	0.806 (0.563–1.154)	0.240
Preoperative eGFR	0.975 (0.950–1.001)	0.064	0.975 (0.948–1.003)	0.082
RENAL score	0.590 (0.431–0.808)	0.001	0.641 (0.455–0.904)	0.011
Surgical type (TRPN vs. RRPN)	1.222 (0.571–2.616)	0.605	1.246 (0.548–2.833)	0.600

OR, odd ratio; CI, confidence interval; BMI, body mass index; CCI, Charlson Comorbidity Index; ASA, American Society of Anesthesiologists; eGFR, estimated glomerular filtration rate; TRPN, transperitoneal robotic partial nephrectomy; RRPN, retroperitoneal robotic partial nephrectomy.

## Discussion

Robotic partial nephrectomy has been more and more widely used in treating renal masses in China and the USA (22), the changing trends in the approach to partial nephrectomy like to the treatment of other genitourinary conditions (23). RPN can be conducted with transperitoneal or retroperitoneal approach (5). The transperitoneal approach is familiar to urologists and adequate operating space to avoid instrument collisions. However, because of the proximity of the abdominal organs, it was more difficult to expose renal hilum and tumors. In contrast, the advantage of the retroperitoneal approach is that it reduces morbidity and speeds recovery by avoiding abdominal and unobstructed access to the hilum (14, 16). Although the comparative study of TRPN and RRPN in renal cancer has been well advanced, relevant data are still needed for the complete upper polar tumor subgroup.

In the present study, we initially compared the perioperative, functional, and oncological outcomes of these two surgical approaches for patients with complete upper pole renal tumors. At first, demographics and disease characteristics were compared between TRPN and RRPN. There were no significant differences in age, gender, BMI, ASA score, CCI score, chronic disease history, tumor size and complexity, and preoperative renal function. Generally, transperitoneal approach was more often chosen for anterior tumors, and retroperitoneal approach was more often chosen for posterior tumors. Analyzing the “A” domain of RENAL score, no significant difference was identified between the TRPN and RRPN groups with regard to anterior (25.0% *vs.* 23.3%), posterior (45.8% *vs.* 41.7%), and not determined (29.2% *vs.* 35.0%, *p* = 0.853). Therefore, there was good comparability between the two groups.

Previously, many studies have compared the outcomes between TRPN and RRPN for patients with renal masses (8–13, 24, 25). Most of them have identified advantages of RRPN, such as shorter operating time and ischemia time, less blood loss, and shorter hospital stay, especially for lateral or posterior tumors. Recently, we have included all studies with good comparability to perform a meta-analysis for this issue. The results showed that RRPN can obtain more favorable outcomes than TRPN, including shorter operative time, less estimated blood loss, less minor complications, and shorter hospital stay (15). However, besides slight advantage in estimated blood loss was found for RRPN, no significant difference was identified for other perioperative, functional, and oncological outcomes. All of the surgical procedures in our study were performed by skilled, high-volume surgeons. Experience with the technique may overcome the disadvantages of each surgical approach. According to our results, RRPN can obtain shorter ischemia time and lower rate of postoperative complications. However, the difference did not obtain statistical significance, which might be due to insufficient sample size. Moreover, the largest study comparing TRPN and RRPN to date reported similar results. After propensity score matching, 768 patients treated with TRPN or RRPN from global multi-institutions were analyzed. RRPN was proven to obtain a less estimated blood loss. No differences were observed between TRPN and RRPN in terms of operative and ischemia time, complications, length of stay, and positive surgical margins (26).

In our study, the perioperative, functional, and oncological outcomes were good, similar to those in other cohorts. Previously, we have reported the outcomes of 603 patients undergoing RPN. The rates of pentafecta achievement and each component were similar to the present study (27). Histopathological examination revealed that most of the patients had malignant tumors. Since there were no differences in tumor size, ischemia time, pT stage, Fuhrman grade, and tumor necrosis, it was understandable that these two approaches achieved comparable renal functional and oncological outcomes. Moreover, the renal functional and oncological outcomes reported in our study were similar to previous literatures (1, 2).

The perioperative outcomes of partial nephrectomy can be affected by many factors (28). As a comprehensive evaluation system, pentafecta achievement was also used to evaluate surgical outcomes of TRPN and RRPN. Because perioperative and functional outcomes were similar in both groups, there were no significant differences between each component. After synthesizing the results of five components, the pentafecta achievement rates of TRPN and RRPN were similar (50.0 *vs.* 55.0%, *p* = 0.699). Logistic regression analyses showed that only decreasing RENAL score (OR, 0.641 (95% CI, 0.455–0.904); *p* = 0.011) was an independent risk factor related to pentafecta achievement, but not surgical type (*p* = 0.600). About this issue, many previous studies have reported relevant results. Stroup et al. (10) have applied pentafecta to compare TRPN and RRPN, and factors related to lack of pentafecta outcome was conducted. The results showed that RENAL score and baseline eGFR were independent risk factors. Choi et al. (9) have analyzed 566 consecutive cases who were treated with RPN by a single surgeon to compare TRPN and RRPN for localized renal masses. Multivariable analysis identified that serum hemoglobin and tumor size were predictors of pentafecta achievement. Sharma et al. (29) have externally validated SPARE score in predicting pentafecta outcomes following RPN; the data of 201 patients undergoing RPN were analyzed. On multivariate analysis, age, preoperative eGFR, and SPARE score were predictors for pentafecta achievement. Different exclusion criteria, study subject, and relevant variables may be partly responsible for the inconsistent results.

There were several limitations for our study. Firstly, the results were limited by retrospective design with single-center inadequate sample size. Inherent selection bias in analyses can be controlled. Nevertheless, the similarity in demographic characteristics and disease features (including tumor complexity) between the two groups suggests their comparability. Secondly, all operations are performed by experienced surgeons. Junior surgeons should interpret these results with caution, choosing of surgical approach should be based on their experience and preference. Despite these limitations, our study initially compared TRPN and RRPN for complete upper pole renal tumors. Demographics, disease characteristics, perioperative outcomes, pathological, renal functional, and oncological outcomes were comprehensively reported and compared.

In summary, TRPN and RRPN can provide good and comparable results in perioperative, function, and oncological outcomes in patients with upper pole renal tumors. Both surgical approaches remain viable options in the treatment of these patients. The choice of surgical approach should be based on relevant experience and the preference of the surgeon.

## Data Availability Statement

The original contributions presented in the study are included in the article/**Supplementary Material**, further inquiries can be directed to the corresponding author.

## Ethics Statement

The studies involving human participants were reviewed and approved by the Ethics Committee of Chinese PLA General Hospital. The patients/participants provided their written informed consent to participate in this study.

## Author Contributions

Study concepts and design: XZ, XM, and LG. Data acquisition: LG, WZ, JX, YX, and XZ. Quality control of data and algorithms: LG, BW, and QC. Data analysis and interpretation: LG, DS, and HL. Statistical analysis: LG, WZ, and JX. Manuscript preparation: LG, WZ, and JX. Manuscript editing: BW and HL. Manuscript review: XZ and XM.

## Funding

This work was supported by the National Natural Science Foundation of China (Grant 82100820).

## Conflict of Interest

The authors declare that the research was conducted in the absence of any commercial or financial relationships that could be construed as a potential conflict of interest.

## Publisher’s Note

All claims expressed in this article are solely those of the authors and do not necessarily represent those of their affiliated organizations, or those of the publisher, the editors and the reviewers. Any product that may be evaluated in this article, or claim that may be made by its manufacturer, is not guaranteed or endorsed by the publisher.
