# Supplementary material for: Comparison of Transperitoneal and Retroperitoneal Robotic Partial Nephrectomy for Patients With Complete Upper Pole Renal Tumors
Source: Front Oncol. 2022 Jan 25;11:773345. doi: 10.3389/fonc.2021.773345 (PMC8821917; doi:10.3389/fonc.2021.773345)

Figure S1. Patient positioning and trocar placement for TRPN.

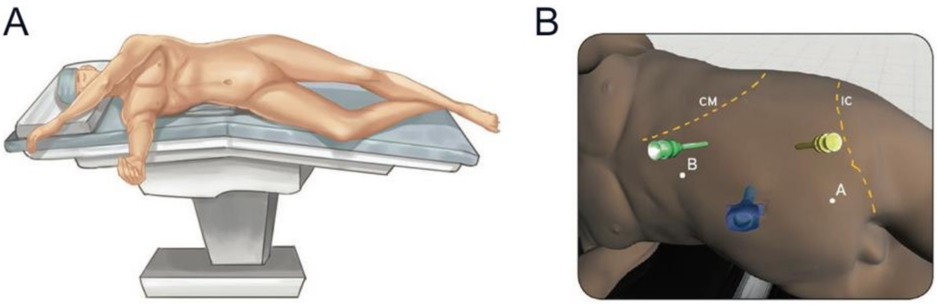

Figure S2. Patient positioning and trocar placement for RRPN.

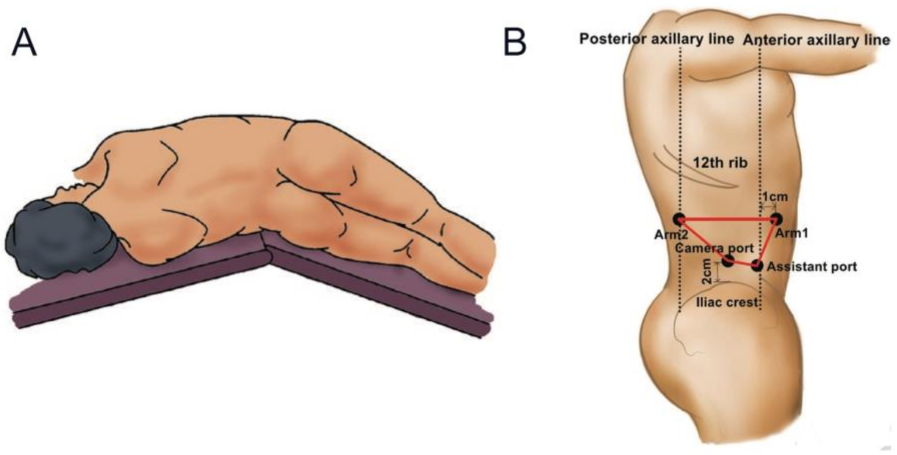

Supplement: Supplementary file 1 [file DataSheet_1.pdf]
